# Immigrant background as a determinant of caries development in Swedish children

**DOI:** 10.1186/s12903-026-09919-7

**Published:** 2026-09-29

**Authors:** Maria Anderson, Anna Oksanen, Maria Chamoun, Gunilla Klingberg, Aron Naimi-Akbar

**Affiliations:** 1 Department of Pediatric Dentistry, Eastman Institute, Public Dental Service, Stockholm, Sweden; 2 Division of Orthodontics and Pediatric Dentistry, Department of Dental Medicine, Karolinska Institutet, Stockholm, Sweden; 3 Center of Pediatric Oral Health Research, Stockholm, Sweden; 4 Department of Medical Epidemiology and Biostatistics, Karolinska Institutet, Stockholm, Sweden; 5 Department of Pediatric Dentistry, Faculty of Odontology, Malmö University, Malmö, Sweden

**Keywords:** Dental caries, Immigrant populations, Longitudinal studies, Preschool children, Risk factors, Sociodemographic determinants of health

## Abstract

**Introduction:**

Inequalities in oral health persist, particularly in highly developed countries, where socioeconomic status is an important explanatory factor. Previous analyses identified immigrant background as a determinant of dental caries development. The present study further explored this association by investigating the influence of home language and the income classification of the country or region of origin on caries development.

**Methods:**

This prospective longitudinal cohort study followed 3,402 children who lived in 23 socioeconomically disadvantaged areas in Stockholm, Sweden, from ages 1 to 3 years. Caries was assessed using the International Caries Detection and Assessment System (ICDAS II) criteria. Primary outcome measures were number of tooth surfaces with dental caries (ICDAS ≥ 1 and ICDAS ≥ 3). Longitudinal data were collected through annual clinical examinations that included questionnaires at ages 1, 2, and 3 years. Immigrant background was defined by the languages spoken at home, which were linked to the income classification of the country or region of origin and categorized as low-, middle-, or high-income European or non-European countries or regions. These factors, home language and income classification, served as exposure variables in the analysis.

**Results:**

The study identified 83 home languages. The reference group, the Swedish language group, had the lowest caries count at all time points and showed the smallest increase in caries development. The Mixed language group (Swedish and one or more foreign home languages) had a caries risk between the reference group (Swedish language) and the non-Swedish language group (no Swedish was spoken at home). Higher caries development was found among some immigrant groups, particularly those from middle-income European countries and non-European countries or regions.

**Conclusion:**

Immigrant background emerged as a sociodemographic determinant, with evident variation in caries outcomes across different groups. Preventive efforts should continue, but further development to better address the specific needs of multicultural, low-socioeconomic communities. Measures such as use of interpreters, multilingual materials, and establishment of community outreach programs could be beneficial.

**Trial registration:**

ISRCTN35086887. Registered 16 December 2013.

**Supplementary Information:**

The online version contains supplementary material available at https://doi.org/10.1186/s12903-026-09919-7.

## Introduction

Anywhere in the world, one of the most common diseases affecting our children is dental caries [1]. In Sweden, dental care is free of charge from birth to 19 years of age [2], and compared with other countries, prevalence of dental caries in Sweden is low at these ages [3]. Still, some children suffer more than others from the consequences of the disease [4]. This is not unique for Sweden, they can be found in other highly developed countries as well, with socioeconomic status being important determinants [5].

In Sweden, poor oral health has been reported in several studies to be more prevalent among children with an immigrant background [6, 7]. Therefore, immigrant background is considered an important determinant for dental caries. In Sweden, about 100,000 children are born each year. The country has become a multicultural society that receives immigrants and refugees from many parts of the world. People from more than 150 countries in the world live here [8]. New arrivals in Sweden often live in socio-economically weaker areas and face unemployment more often than Swedes and established immigrants. Statistics show that it takes around 10 years for immigrants to attain similar living conditions as the rest of the population [9].

These socioeconomic circumstances [10], and the life-style habits that new immigrants adopt in the receiving country or bring with them from their home country, might explain the differences in general and oral health between immigrant groups and the general Swedish population. Adair et al. showed that parental attitudes have an impact on the habits advantageous to oral health and that these habits vary with cultural and ethnic background [11]. A higher consumption of sugar and a lower frequency of tooth brushing and use of fluoride toothpaste have been observed among children with a foreign background [12, 13].

Julihn et al. found that, besides the socioeconomic position of the family in Sweden, the dental health of immigrant children could be reflected in the developmental level of the parental country of birth [14]. Among children with an immigrant background, limited knowledge exists regarding the factors associated with developing caries or remaining caries free.

Our study is based on a large cohort of 1-year-olds born in 2010 in socioeconomically disadvantaged areas of Stockholm [15], followed longitudinally. Language spoken at home and dental care habits were reported directly by the parents, allowing us to capture new aspects of the influence of immigration and other cultural factors on children’s dental health. 80% of the children had an immigrant background, which was also identified as a determinant of dental caries [16, 17]. An economic evaluation of the same study observed that the utilization of interpreters was limited, accounting for only 0.37–1.6% of the visits. This may be explained by the fact that staff members speak many of the most common languages represented, which may lead to such information not being consistently reported in dental records [18]. Language can act as a barrier in health care access [19]. Given that oral health literacy involves accessing, understanding, and applying health information to make informed decisions [20], it is crucial to recognize that dental health promotion and preventive measures rely on effective communication, including dialogue and tailored instructions.

With these findings in mind and the recurring association in the literature between oral health inequalities and immigrant background, we aimed to explore the background characteristics of these children in greater detail to better understand variations in oral health outcomes. The aim of this study was to examine immigrant background as a sociodemographic determinant in greater depth by analyzing how the language spoken at home and the income level of the country of origin (categorized as low, middle, or high income, and as European or non-European) could influence the dental caries development in young children.

## Materials and methods

### Study design and setting

This is a prospective, longitudinal cohort study based on data from children who participated in the Swedish prospective, cluster-randomized intervention trial “Stop Caries Stockholm” (SCS) [16]. Twenty-three dental clinics in multicultural areas in Stockholm participated in the intervention trial between March 2011 and March 2014. The socioeconomic status of the areas was medium to low. The Regional Ethics Committee in Stockholm previously approved the SCS trial (Daybook no. [Dnr] 2010/1956) and an updated study protocol was approved by the Swedish Ethical Review Authority (Daybook no. 2023/00738 − 302).

### Study population

The study population comprised 1-year-old children born in 2010. All eligible children were invited to participate, and their parents or guardians signed informed-consent forms allowing their children to participate in the study. The SCS intervention trial compared two fluoride based dental caries interventions for preschoolers over two years, with no significant difference in caries development [16].

### Data collection

Longitudinal data were collected at the annual visits when the children were 1, 2, and 3 years old. The data consisted of results from the clinical examination and information obtained through a caregiver‑completed questionnaire. The questionnaire was filled out during an interview conducted by the examiner with the parent or guardian. During the interview, the examiner collected information on socioeconomic conditions, the language spoken at home, and dietary and toothbrushing habits, recording all responses on the survey questionnaire [21].

### Clinical examinations

Clinical data on dental caries were interpreted according to the revised International Caries Detection and Assessment System (ICDAS II) to determine the development stage of dental caries [22]. In Sweden both dentists and dental hygienists are authorized to diagnose dental caries. We trained 49 examiners (24 dental hygienists, 25 dentists) from public dental clinics in ICDAS II scoring. Training included a 90-minute e-learning module [23], followed by a half-day lecture with calibration exercises using an audience response system. To assess reliability, all examiners scored 25 images of carious tooth surfaces (ICDAS II scores 0–6). Agreement with a reference standard (two experienced examiners) yielded a mean kappa of 0.66 (range 0.38–1). When using score 3 as the cutoff (ICDAS II ≥ 3), the mean kappa increased to 0.85 (range 0.48–1).

Clinical reliability was further evaluated through examinations of 20 children aged 3 to 5 years. One experienced examiner and one newly trained examiner independently assessed caries using ICDAS II scores, with re-examinations conducted two weeks later. Intraexaminer reliability was κ = 0.62 for the experienced examiner and κ = 0.49 for the newly trained examiner. Interexaminer reliability was κ = 0.42 during the first examination and κ = 0.60 during the second. When using score 3 as the cutoff, intraexaminer reliability was κ = 0.72 and κ = 0.73, and interexaminer reliability was κ = 0.61 and κ = 0.73 for the first and second examinations, respectively.

These findings indicate moderate to substantial agreement, with improved reliability when focusing on more advanced lesions (ICDAS II ≥ 3). For more information, see Anderson et al. [16] and the Karolinska Institutet website [21].

### Outcome measures

The two outcome measures in the present study were (1) number of tooth surfaces with initial or more severe caries (a tooth surface with an ICDAS score of 1 or higher, ICDAS ≥ 1) and (2) number of tooth surfaces with localized enamel breakdown due to caries, but with no visible dentine, or more severe (a tooth surface with an ICDAS score of 3 or higher, ICDAS ≥ 3).

### Model 1: Home language

Model 1 used only one exposure variable, derived from the questionnaire: home language (the language spoken at home). The study cohort was divided into three groups: Swedish, Mixed, and Non-Swedish. If only Swedish was spoken at home, the child was placed in the Swedish group. If Swedish *and* one or more languages were spoken at home, the child was placed in the Mixed languages group. If no Swedish and only foreign languages were spoken at home, the child was placed in the “Non-Swedish“ language group.

### Model 2: Home language, and income classification of the region or country of origin – grouping I 

Model 2 introduced a second exposure variable: income classification of the region or country of origin. We first created five categories, based on the questionnaire data. The countries represented in the study cohort were classified as European or non-European. Income classification of the country was designated as low-, middle- or high-income based on the World Bank calculations of gross national income per capita [24]. We inferred which country the family had immigrated from based on the language spoken at home [25, 26]. Languages that could not be linked to a country were linked to a geographical region. Some families spoke several languages at home. In this case, we grouped children based on the non-Swedish language.

Thus, Model 2 grouped the children according to their home language, and in the case of non-Swedish home languages, to the income classification of their region or country of origin. Six categories were defined: Sweden (the only home language was Swedish); and High-income European country, Middle-income European country, High-income non-European country, Middle-income non-European country, and Low-income non-European country (the home language was not Swedish, or the home languages were other languages, one of which could be Swedish) [24]. Supplement 1 presents these groups.

### Model 3: Home language, and income classification of the region or country of origin – grouping II 

Model 3 had seven categories, also based on home language and on the region or country of origin and its income classification. The aim of the groupings in this model, however, was to discern whether speaking Swedish in a multi-lingual home was a significant factor. Model 3 adopted the first two groups in Model 1: Swedish (only Swedish was spoken at home) and Mixed (Swedish and one other language were spoken at home). The five region and income classification groups in Model 2 were then redefined: only children who spoke one or more foreign languages, but no Swedish, at home were placed in the five region and income classification categories.

### Statistical analysis

Continuous variables were summarized using means and standard deviations, whereas categorical variables were presented as counts and percentages. Because most children had no caries at this early age, the caries counts for ICDAS ≥ 1 and ICDAS ≥ 3 were heavily zero‑inflated. To account for this distributional feature, a zero‑inflated negative binomial model was applied. This model consists of two components: a logistic regression estimating the probability of an excess zero, and a negative binomial component estimating the expected number of caries‑affected tooth surfaces among children whose values were not excess zeros. Although the excess zeros may reflect children in whom no caries had yet developed, this conceptual interpretation is not necessary for the statistical model to remain valid.

The model used was a mixed effects zero-inflated negative binomial model with a person-specific random intercept. A mixed model was used to account for the non-independence of observations in repeated measures. In addition to the random intercept, the negative binomial model included a term for age (1, 2, or 3 years) and the language group, where monolingual Swedish was always the reference group. Three groupings based on the home language were used: first with Swedish, Mixed, and non-Swedish only; second with six groups including only Swedish and non-Swedish, and third with seven groups, which included Swedish, non-Swedish, and the Mixed group.

Besides the primary analysis, two secondary adjusted analyses were performed. The first of these adjusted for tooth brushing habits as well as consumption of candy and sweet drinks. The second adjusted for maternal education level and family income. All adjusted variables were categorical. The zero-inflation model estimated the time effect without interactions. Graphical presentation of the estimated differences was based on the marginal means. Analysis was done using R, version 4.1.0 [27]. The negative binomial model was estimated with the R package “glmmTMB” [28], and marginal means were estimated and graphically presented with the help of “ggeffects” [29].

## Results

**Fig. 1:**
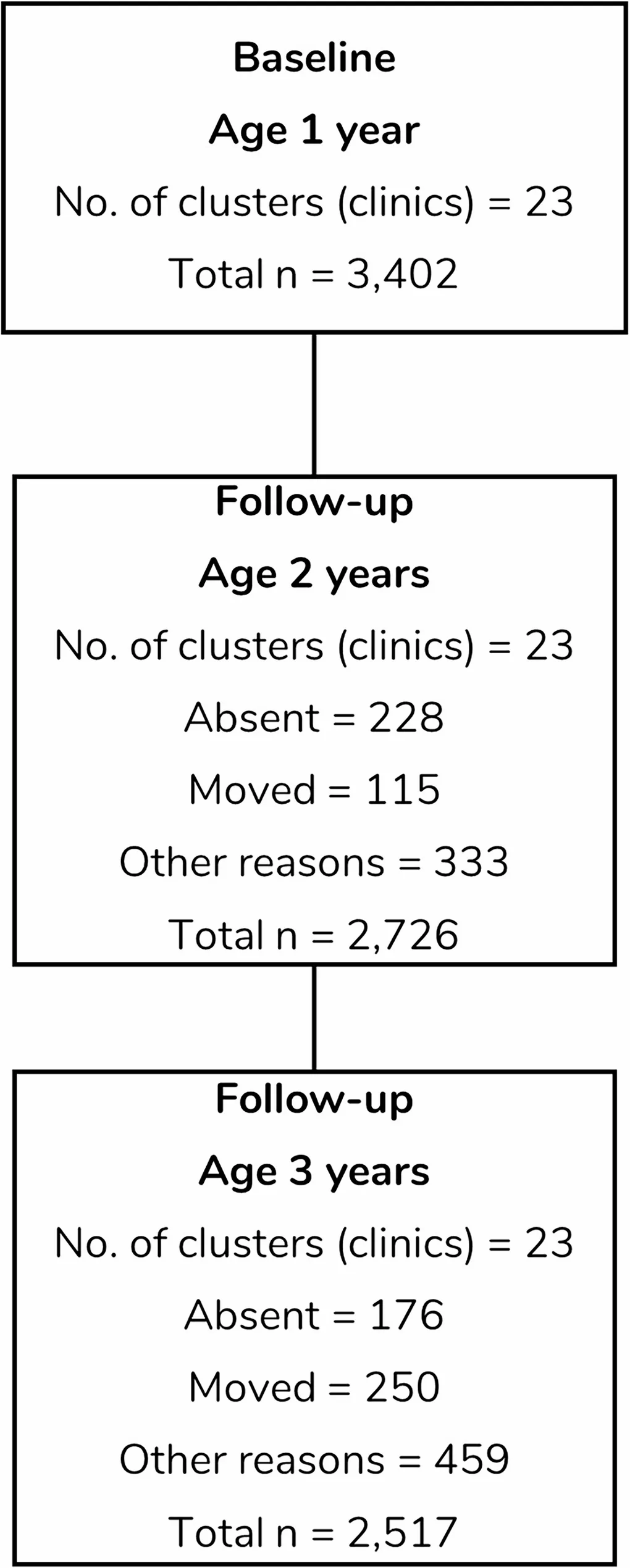
Flowchart. Flowchart showing all children and dental clinics (clusters) included at 1 year, with retention and reason for dropout at 2 and 3 years

At baseline, 3,402 1-year-old children visited the dental clinics and were included in the study. At age 2, 78.6% (*n* = 2,675) of the baseline group showed up for their examination, the first follow-up, and continued to be included in the study. At age 3 years, 74.5% (*n* = 2,536) of the baseline group showed up for the final follow-up. Fig. 1 presents the reasons for dropping out at the follow-ups. Differences in attendance rates between the three age groups were non-significant.

Table 1 presents the baseline characteristics as well as the number of subjects in the three age groups. The groups were not evenly distributed, reflecting the characteristics of the underlying dataset. The largest group was Middle-Income non-European country with 1,190 subjects (35.0%), the Swedish group comprised 726 subjects (21.3%), and the Mixed group, 618 (18.2%). In all, participants in the study cohort spoke 83 languages. The most common non-Swedish language was Arabic followed by Kurdish, Turkish, Somali, and Polish. Tables 2, 3 and 4 present the participant characteristics in the country of origin categories at ages 1, 2 and 3 years respectively.

**Table 1:** Country of origin categories, home language categories, and dental status at 1, 2 and 3 years of age

Contry of origin category	Home language category	Children 1 year (*n* = 3402)			Children 2 years (*n* = 2726)			Children 3 years (*n* = 2517)		
		Caries-free	ICDAS *≥* 1	ICDAS *≥* 3	Caries-free	ICDAS *≥* 1	ICDAS *≥* 3	Caries-free	ICDAS *≥* 1	ICDAS *≥* 3
Sweden	Swedish*	715 (98.48%)	11 (1.52%)	1 (0.14%)	568 (95.14%)	29 (4.86%)	6 (1.01%)	459 (87.60%)	65 (12.40%)	22 (4.20%)
Mixed	Mixed**	583 (94.34%)	35 (5.66%)	5 (0.81%)	428 (87.53%)	61 (12.47%)	21 (4.29%)	366 (81.15%)	85 (18.85%)	48 (10.64%)
	Non-Swedish***									
High income European country	Croatian, Danish, Dutch, English, Estonian, Finnish, French, German, Greek, Hungarian, Icelandic, Italian, Latvian, Lithuanian, Norwegian, Polish, Portuguese, Romani, Romanian, Spanish	339 (94.43%)	20 (5.57%)	2 (0.56%)	242 (85.82%)	40 (14.18%)	14 (4.96%)	204 (77.57%)	59 (22.43%)	36 (13.69%)
Minddle income European country	Albanian, Bosnian, Bulgarian, Macedonian, Montenegrin, Russian, Serbian, Serbo-Croatian, Ukrainian	122 (93.13%)	9 (6.87%)	1 (0.76%)	88 (83.81%)	17 (16.19%)	9 (8.57%)	69 (71.13%)	28 (28.87%)	18 (18.56%)
High income non European country	Cantonese, Chinese, Japanese, Mandarin, Uyghur	18 (81.82%)	4 (18.18%)	1 (4.55%)	11 (64.71%)	6 (35.29%)	3 (17.65%)	12 (66.67%)	6 (33.33%)	5 (27.78%)
Middle income non European country	Filipino, Indonesian, Tagalog, Thai, Armenian, Kurdish, Mongolian, Turkish, Turkmen, Uzbek, Vietnamese, Afrikaans, Akan/Twi, Berber, Fula, Igbo, Kikuyu, Yoruba, Bengali, Gujarati, Hindi, Nepali, Punjabi, Rohingya, Sinala, Tamil, Telugu, Urdu, Arabic, Aramaic, Assyrian, Chaldean, Persian, Suryoyo, Syriac	1109 (93.19%)	81 (6.81%)	12 (1.01%)	793 (82.01%)	174 (17.99%)	65 (6.72%)	627 (69.05%)	281 (30.95%)	161 (17.73%)
Low income non European country	Amharic, Bantu, Gambian English, Kirundi, Lingala, Mandinka, Somali, Soninke, Swahili, Tigrinia, Wolof, Dari, Pashto	339 (95.22%)	17 (4.78%)	0 (0%)	224 (83.27%)	45 (16.73%)	10 (3.72%)	187 (73.05%)	69 (26.95%)	40 (15.62%)

* Only Swedish spoken at home, ** Swedish and other languages spoken at home *** No Swedish, only languages other than Swedish spoken at home

**Table 2:** Participant characteristics (independent variables) of the study population included in the baseline statistical analyses: age 1 year

Characteristic	Sweden* n = 726	Mixed** n = 618	High income European country*** n = 359	Middle income European country*** n = 131	High income non-European country*** n = 22	Middle income non-European country*** n = 1190	Low income non-European country*** n = 356	Total, *n* = 3402
Gender								
Male	380 (52%)	308 (50%)	166 (46%)	61 (47%)	10 (45%)	543 (46%)	174 (49%)	1642 (48%)
Female	345 (48%)	310 (50%)	191 (54%)	69 (53%)	12 (55%)	645 (54%)	182 (51%)	1754 (52%)
Missing	1	0	2	1	0	2	0	6
Mother’s education								
≤ 9 years	46 (6.4%)	92 (15%)	37 (10%)	11 (8.5%)	8 (36%)	362 (31%)	165 (49%)	721 (21%)
10–12 years	314 (44%)	290 (47%)	145 (41%)	64 (49%)	5 (23%)	449 (38%)	113 (33%)	1380 (41%)
> 12 years	359 (50%)	231 (38%)	173 (49%)	55 (42%)	9 (41%)	370 (31%)	62 (18%)	1259 (37%)
Missing	7	5	4	1	0	9	16	42
Family monthly income								
≤ 20.000 SEK	79 (11%)	202 (33%)	134 (38%)	42 (32%)	11 (50%)	644 (56%)	194 (58%)	1,306 (40%)
21–30.000 SEK	167 (23%)	207 (34%)	136 (39%)	58 (45%)	5 (23%)	342 (30%)	102 (31%)	1,017 (31%)
> 30.000 SEK	466 (65%)	197 (33%)	81 (23%)	30 (23%)	6 (27%)	158 (14%)	38 (11%)	976 (30%)
Missing	14	12	8	1	0	46	22	103
Toothbrushing frequency								
Never	66 (9.2%)	175 (29%)	111 (31%)	60 (46%)	14 (64%)	583 (49%)	140 (40%)	1149 (34%)
A few times a week	72 (10%)	59 (9.6%)	34 (9.6%)	12 (9.2%)	2 (9.1%)	153 (13%)	23 (6.6%)	355 (11%)
1 time/day	179 (25%)	163 (27%)	79 (22%)	32 (24%)	2 (9.1%)	238 (20%)	56 (16%)	749 (22%)
2 times/day	396 (55%)	214 (35%)	125 (35%)	27 (21%)	4 (18%)	197 (17%)	127 (37%)	1090 (32%)
>2 times/day	6 (0.8%)	3 (0.5%)	6 (1.7%)	0 (0%)	0 (0%)	11 (0.9%)	1 (0.3%)	27 (0.8%)
Missing	7	4	4	0	0	8	9	32
Consumption of sugar-containing beverages								
Never	332 (46%)	247 (40%)	114 (32%)	51 (39%)	9 (41%)	395 (33%)	107 (31%)	1255 (37%)
A few times a week	177 (25%)	183 (30%)	96 (27%)	29 (22%)	7 (32%)	408 (34%)	87 (25%)	987 (29%)
1 time/day	88 (12%)	65 (11%)	69 (19%)	20 (15%)	4 (18%)	140 (12%)	51 (15%)	437 (13%)
2 times/day	72 (10.0%)	56 (9.1%)	41 (12%)	15 (11%)	0 (0%)	97 (8.2%)	45 (13%)	326 (9.7%)
>2 times/day	52 (7.2%)	63 (10%)	36 (10%)	16 (12%)	2 (9.1%)	145 (12%)	59 (17%)	373 (11%)
Missing	5	4	3	0	0	5	7	24
Candy consumption								
Never	617 (85%)	437 (71%)	227 (64%)	86 (66%)	14 (64%)	741 (62%)	260 (74%)	2382 (70%)
1 time/week	83 (11%)	130 (21%)	94 (26%)	29 (22%)	3 (14%)	286 (24%)	78 (22%)	703 (21%)
A few times a week	21 (2.9%)	45 (7.3%)	31 (8.7%)	13 (9.9%)	4 (18%)	130 (11%)	9 (2.6%)	253 (7.5%)
Every day	2 (0.3%)	3 (0.5%)	4 (1.1%)	3 (2.3%)	1 (4.5%)	30 (2.5%)	3 (0.9%)	46 (1.4%)
Missing	3	3	3	0	0	3	6	18
Gingivitis								
Not present	677 (96%)	578 (95%)	330 (94%)	121 (95%)	16 (76%)	1,110 (95%)	339 (97%)	3171 (95%)
Present	27 (3.8%)	33 (5.4%)	20 (5.7%)	7 (5.5%)	5 (24%)	58 (5.0%)	10 (2.9%)	160 (4.8%)
Missing	22	7	9	3	1	22	7	71

* Only Swedish spoken at home, ** Swedish and other languages spoken at home, *** Other language than Swedish spoken at home

**Table 3:** Participant characteristics (independent variables) of the study population included in the statistical analyses: age 2 years

Characteristic	Sweden* n = 524	Mixed** n = 451	High income European country*** n = 263	Middle income European country*** n = 97	High income non-European country*** n = 18	Middle income non-European country*** n = 908	Low income non-European country*** n = 256	Total, *n* = 2517
Gender								
Male	270 (52%)	231 (51%)	122 (46%)	40 (41%)	8 (44%)	424 (47%)	118 (46%)	1213 (48%)
Female	254 (48%)	220 (49%)	141 (54%)	57 (59%)	10 (56%)	483 (53%)	138 (54%)	1303 (52%)
Missing	0	0	0	0	0	1	0	1
Mother’s education								
≤ 9 years	34 (6.5%)	67 (15%)	30 (11%)	8 (8.3%)	7 (39%)	285 (32%)	127 (52%)	558 (22%)
10–12 years	234 (45%)	209 (47%)	109 (42%)	51 (53%)	5 (28%)	330 (37%)	83 (34%)	1021 (41%)
> 12 years	254 (49%)	172 (38%)	122 (47%)	37 (39%)	6 (33%)	285 (32%)	36 (15%)	912 (37%)
Missing	2	3	2	1	0	8	10	26
Family monthly income								
≤ 20.000 SEK	54 (10%)	152 (34%)	97 (37%)	29 (30%)	10 (56%)	489 (56%)	137 (57%)	968 (40%)
21–30.000 SEK	114 (22%)	146 (33%)	104 (40%)	47 (48%)	4 (22%)	271 (31%)	76 (32%)	762 (31%)
> 30.000 SEK	349 (68%)	144 (33%)	58 (22%)	21 (22%)	4 (22%)	115 (13%)	27 (11%)	718 (29%)
Missing	7	9	4	0	0	33	16	69
Toothbrushing frequency								
Never	0 (0%)	4 (0.9%)	3 (1.2%)	1 (1.1%)	0 (0%)	12 (1.3%)	1 (0.4%)	21 (0.8%)
A few times a week	9 (1.7%)	33 (7.3%)	10 (4.0%)	6 (6.3%)	1 (5.9%)	105 (12%)	3 (1.2%)	167 (6.7%)
1 time/day	78 (15%)	106 (24%)	49 (19%)	27 (28%)	4 (24%)	314 (35%)	41 (16%)	619 (25%)
2 times/day	426 (82%)	301 (67%)	178 (71%)	58 (61%)	12 (71%)	455 (51%)	197 (79%)	1627 (66%)
>2 times/day	6 (1.2%)	5 (1.1%)	12 (4.8%)	3 (3.2%)	0 (0%)	14 (1.6%)	7 (2.8%)	47 (1.9%)
Missing	5	2	11	2	1	8	7	36
Consumption of sugar-containing beverages								
Never	234 (45%)	175 (39%)	82 (31%)	38 (39%)	8 (44%)	293 (32%)	76 (30%)	906 (36%)
A few times a week	132 (25%)	135 (30%)	72 (28%)	18 (19%)	6 (33%)	311 (34%)	60 (24%)	734 (29%)
1 time/day	68 (13%)	47 (10%)	51 (20%)	15 (15%)	2 (11%)	106 (12%)	40 (16%)	329 (13%)
2 times/day	49 (9.4%)	42 (9.4%)	31 (12%)	14 (14%)	0 (0%)	83 (9.2%)	31 (12%)	250 (10.0%)
>2 times/day	40 (7.6%)	49 (11%)	25 (9.6%)	12 (12%)	2 (11%)	111 (12%)	45 (18%)	284 (11%)
Missing	1	3	2	0	0	4	4	14
Candy consumption								
Never	74 (14%)	40 (8.9%)	28 (11%)	8 (8.4%)	2 (12%)	61 (6.8%)	27 (11%)	240 (9.7%)
1 time/week	382 (74%)	294 (65%)	146 (57%)	34 (36%)	8 (47%)	493 (55%)	192 (77%)	1,549 (62%)
A few times a week	61 (12%)	112 (25%)	75 (30%)	49 (52%)	7 (41%)	314 (35%)	28 (11%)	646 (26%)
Every day	1 (0.2%)	3 (0.7%)	5 (2.0%)	4 (4.2%)	0 (0%)	31 (3.4%)	2 (0.8%)	46 (1.9%)
Missing	6	2	9	2	1	9	7	36
Gingivitis								
Not present	497 (98%)	421 (94%)	239 (93%)	92 (96%)	12 (71%)	845 (95%)	247 (97%)	2,353 (95%)
Present	12 (2.4%)	25 (5.6%)	17 (6.6%)	4 (4.2%)	5 (29%)	47 (5.3%)	7 (2.8%)	117 (4.7%)
Missing	15	5	7	1	1	16	2	47

* Only Swedish spoken at home, ** Swedish and other language spoken at home, *** No Swedish, only languages other than Swedish spoken at home

**Table 4:** Participant characteristics (independent variables) of the study population included in the statistical analyses: age 3 years

Characteristic	Sweden* n = 524	Mixed** n = 451	High income European country*** n = 263	Middle income European country*** n = 97	High income non-European country*** n = 18	Middle income non-European country*** n = 908	Low income non-European country*** n = 256	Total, *n* = 2517
Gender								
Male	270 (52%)	231 (51%)	122 (46%)	40 (41%)	8 (44%)	424 (47%)	118 (46%)	1213 (48%)
Female	254 (48%)	220 (49%)	141 (54%)	57 (59%)	10 (56%)	483 (53%)	138 (54%)	1303 (52%)
Missing	0	0	0	0	0	1	0	1
Mother’s education								
≤ 9 years	34 (6.5%)	67 (15%)	30 (11%)	8 (8.3%)	7 (39%)	285 (32%)	127 (52%)	558 (22%)
10–12 years	234 (45%)	209 (47%)	109 (42%)	51 (53%)	5 (28%)	330 (37%)	83 (34%)	1021 (41%)
> 12 years	254 (49%)	172 (38%)	122 (47%)	37 (39%)	6 (33%)	285 (32%)	36 (15%)	912 (37%)
Missing	2	3	2	1	0	8	10	26
Family monthly income								
≤ 20.000 SEK	54 (10%)	152 (34%)	97 (37%)	29 (30%)	10 (56%)	489 (56%)	137 (57%)	968 (40%)
21–30.000 SEK	114 (22%)	146 (33%)	104 (40%)	47 (48%)	4 (22%)	271 (31%)	76 (32%)	762 (31%)
> 30.000 SEK	349 (68%)	144 (33%)	58 (22%)	21 (22%)	4 (22%)	115 (13%)	27 (11%)	718 (29%)
Missing	7	9	4	0	0	33	16	69
Toothbrushing frequency								
Never	0 (0%)	4 (0.9%)	3 (1.2%)	1 (1.1%)	0 (0%)	12 (1.3%)	1 (0.4%)	21 (0.8%)
A few times a week	9 (1.7%)	33 (7.3%)	10 (4.0%)	6 (6.3%)	1 (5.9%)	105 (12%)	3 (1.2%)	167 (6.7%)
1 time/day	78 (15%)	106 (24%)	49 (19%)	27 (28%)	4 (24%)	314 (35%)	41 (16%)	619 (25%)
2 times/day	426 (82%)	301 (67%)	178 (71%)	58 (61%)	12 (71%)	455 (51%)	197 (79%)	1627 (66%)
>2 times/day	6 (1.2%)	5 (1.1%)	12 (4.8%)	3 (3.2%)	0 (0%)	14 (1.6%)	7 (2.8%)	47 (1.9%)
Missing	5	2	11	2	1	8	7	36
Consumption of sugar-containing beverages								
Never	234 (45%)	175 (39%)	82 (31%)	38 (39%)	8 (44%)	293 (32%)	76 (30%)	906 (36%)
A few times a week	132 (25%)	135 (30%)	72 (28%)	18 (19%)	6 (33%)	311 (34%)	60 (24%)	734 (29%)
1 time/day	68 (13%)	47 (10%)	51 (20%)	15 (15%)	2 (11%)	106 (12%)	40 (16%)	329 (13%)
2 times/day	49 (9.4%)	42 (9.4%)	31 (12%)	14 (14%)	0 (0%)	83 (9.2%)	31 (12%)	250 (10.0%)
>2 times/day	40 (7.6%)	49 (11%)	25 (9.6%)	12 (12%)	2 (11%)	111 (12%)	45 (18%)	284 (11%)
Missing	1	3	2	0	0	4	4	14
Candy consumption								
Never	74 (14%)	40 (8.9%)	28 (11%)	8 (8.4%)	2 (12%)	61 (6.8%)	27 (11%)	240 (9.7%)
1 time/week	382 (74%)	294 (65%)	146 (57%)	34 (36%)	8 (47%)	493 (55%)	192 (77%)	1,549 (62%)
A few times a week	61 (12%)	112 (25%)	75 (30%)	49 (52%)	7 (41%)	314 (35%)	28 (11%)	646 (26%)
Every day	1 (0.2%)	3 (0.7%)	5 (2.0%)	4 (4.2%)	0 (0%)	31 (3.4%)	2 (0.8%)	46 (1.9%)
Missing	6	2	9	2	1	9	7	36
Gingivitis								
Not present	497 (98%)	421 (94%)	239 (93%)	92 (96%)	12 (71%)	845 (95%)	247 (97%)	2,353 (95%)
Present	12 (2.4%)	25 (5.6%)	17 (6.6%)	4 (4.2%)	5 (29%)	47 (5.3%)	7 (2.8%)	117 (4.7%)
Missing	15	5	7	1	1	16	2	47

* Only Swedish spoken at home, ** Swedish and other language spoken at home, *** No Swedish, only languages other than Swedish spoken at home

### Dental caries

At baseline, 177 (5.2%) subjects were diagnosed with dental caries (ICDAS ≥ 1). At the first annual follow-up it had increased to 372 (13.6%), and by the end of the study at the second annual follow-up, to 593 (23.6%). At baseline, 22 (0.6%) subjects were diagnosed with dental caries (ICDAS ≥ 3). At age 2 years ICDAS ≥ 3 had increased to 128 (4.7%), and by the end of the study, to 330 (13.1%).

**Fig. 2:**
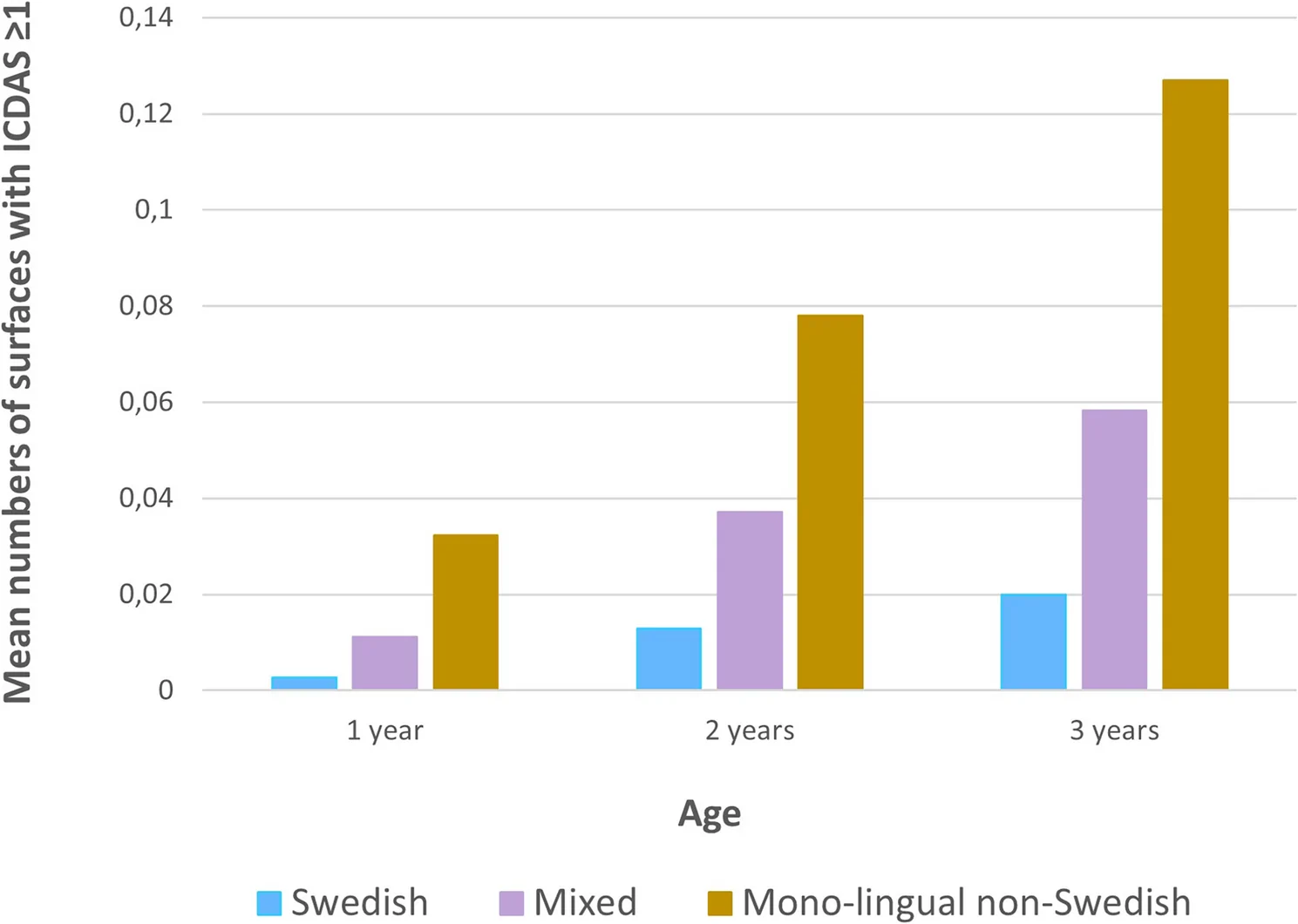
Model 1: Home language. The predicted mean number of dental surfaces having a classification according to the International Caries Detection and Assessment System (ICDAS II) of ICDAS ≥ 1. Model 1 grouped the study cohort into three groups based on home language alone. The Swedish group differed significantly from the other two groups (*p* < 0.001). Differences between the Mixed and Non-Swedish groups were not significant (*p* = 0.41)

**Fig. 3:**
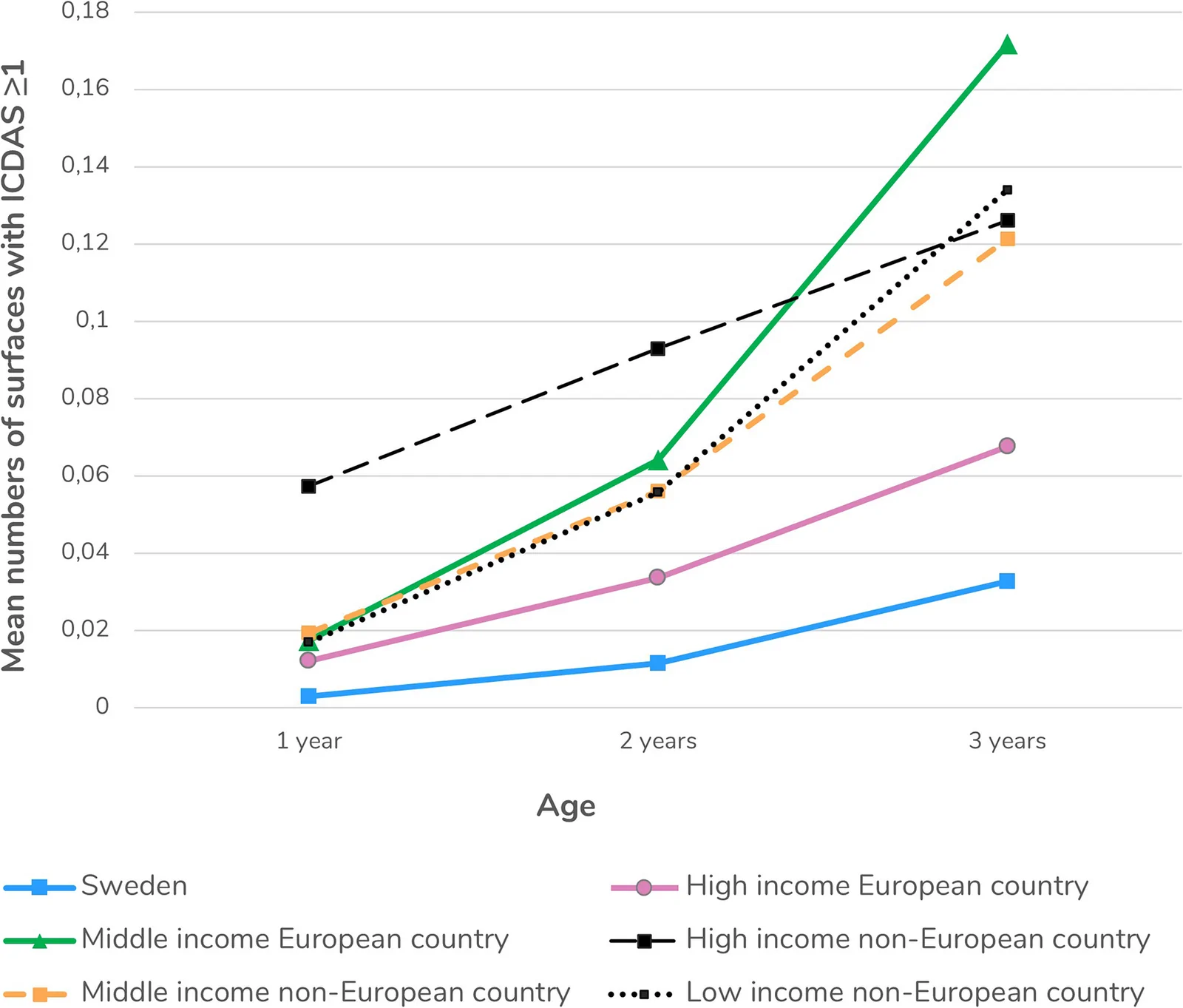
Model 2: Home language and region or country of origin and its income classification – grouping I. The predicted mean number of dental surfaces having a classification according to the International Caries Detection and Assessment System (ICDAS II) of ICDAS ≥ 1. Model 2 grouped the study cohort into six groups based on home language and on region or country of origin and its income classification. In the five groups of children with a non-Swedish language spoken at home, was included in the income classification of the region of origin. The Sweden group differed significantly from the other five groups (*p* < 0.001)

**Fig. 4:**
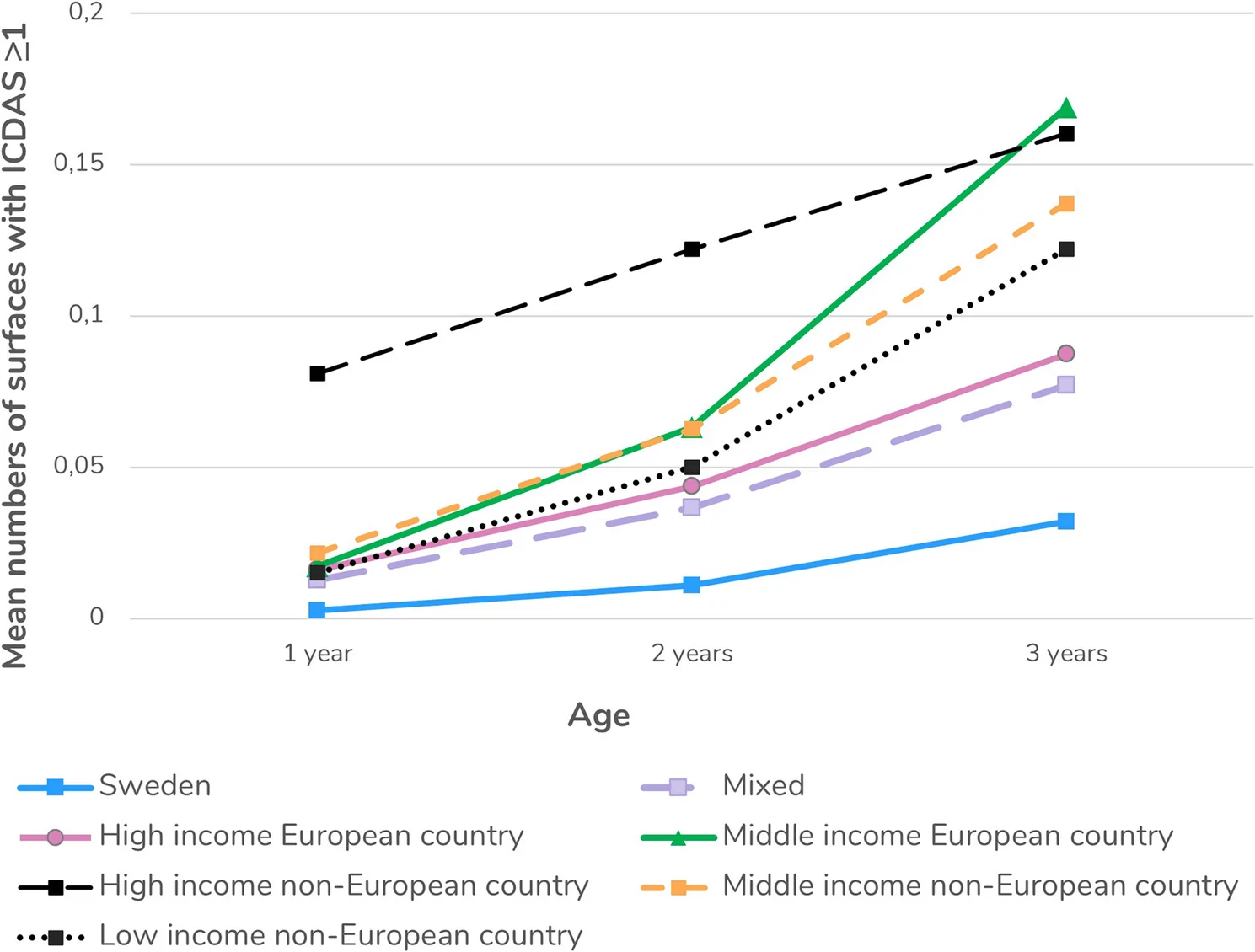
Home language and region or country of origin and its income classification – grouping II. The predicted mean number of dental surfaces having a classification according to the International Caries Detection and Assessment System (ICDAS II) of ICDAS ≥ 1. Model 3 grouped the study cohort into seven groups, adopting the first two groups in Model 1 (Swedish and Mixed) and redefining the five income classification groups that were introduced in Model 2: children in multilingual homes where Swedish was one of the home languages were placed in the Mixed group. The Sweden group differed significantly from the other six groups (*p* < 0.001)

Figures 2, 3 and 4 present the predicted means for ICDAS ≥ 1 in the zero-inflated negative binomial model for the three study models at baseline and at the two follow-ups. The mean number of surfaces with dental caries was small in all groups, as expected. Zero inflation diminishes for each time point in all the models.

The group that spoke only Swedish at home had the lowest caries counts at all examinations, exhibiting the flattest increase. Table 5 presents the regression coefficients for “Model 1 for home language”. Caries increase in the Mixed language group was higher than in the Swedish reference group but lower than in the other groups, as Figs. 2, 3 and 4 show. The pattern looked similar for both outcome measures (ICDAS ≥ 1 and ICDAS ≥ 3). The models were adjusted for tooth brushing habits, consumption of candy and sweet drinks, maternal education level, and family income. The highest education level as well as higher income level were significantly associated with a decrease in dental caries (ICDAS ≥ 1 and ICDAS ≥ 3). Sugar-containing beverages were significantly associated with an increase in dental caries as well as eating candy every day. Supplement 2 presents all the remaining regression coefficients.

**Table 5:** Results from a mixed-effects zero-inflated negative binomial model with a person-specific random intercept for the longitudinal analysis of ICDAS II ≥ 1. The exposure variable comprised three categories. Regression coefficients (β), standard errors (SE), and corresponding p-values are presented. Predicted annual means in caries count for the different exposure groups are also shown

Fixed effects model			
	Value	Std.Error	*p*-value
(Intercept)	-5.3981	0.1950	< 0.0001
Dummy-No Swedish	1.9278	0.2092	< 0.0001
Dummy-Mixed	1.7909	0.2455	< 0.0001
time	0.7228	0.0518	< 0.0001
Dummy-No Swedish*time	-0.2067	0.0532	11,004
Dummy-Mixed*time	-0.3079	0.0583	< 0.0001

Zero-inflation model			
(Intercept)	2.0547		
Dummy-No Swedish	-0.3854		
Dummy-Mixed	-0.0794		
time	-1.1839		

Predicted mean caries count in the different groups			
	Dummy-	time	predicted
	Mixed	1	0.0128
	Mixed	2	0.0371
	Mixed	3	0.0781
	No Swedish	1	0.0199
	No Swedish	2	0.0583
	No Swedish	3	0.1270
	Swedish	1	0.0028
	Swedish	2	0.0111
	Swedish	3	0.0323

## Discussion

This study examined the language spoken at home and the income level of the country of origin, categorized as low, middle, or high income and as European or non‑European, is associated with the development of dental caries in young children, with the overall aim of gaining a deeper understanding of immigrant background as sociodemographic determinant for dental caries.

The study revealed a highly multilingual environment, reflecting the diverse cultural composition of the cohort in caries high‑risk areas. Immigrant background emerged as a sociodemographic determinant, with evident variation in caries outcomes across different groups. The analyses demonstrated higher dental caries development among children who spoke no Swedish at home, home countries outside Europe, and those originating from European countries with middle income economies.

Not speaking Swedish at home, which accounted for 60% of the children, could indicate a relatively recent arrival in Sweden. Alternatively, other factors, such as the desire to maintain cultural continuity with their country of origin and heritage by speaking their native language, are a common aspect of transnationalism [30]. The children who spoke both Swedish and another language at home had lower predicted development of dental caries than those who did not report speaking Swedish at all. Language has been suggested to serve as a proxy method for measuring acculturation [31].

This suggests that our findings are in line with a systematic review of 42 quantitative studies conducted across different regions of the world. These studies often used language factors and length of stay as indicators of acculturation and found these factors to be associated with improved oral health outcomes [32]. In the 3-year-old group, the largest predicted caries counts were observed among children from Middle-Income European countries. This finding aligns with results from another Swedish study that also reported higher dental caries prevalence in children whose mothers came from countries with a moderate economic development [33]. One possible explanation for this finding in our study could be the relatively high frequency of candy consumption within this group: 56% of these children reported eating candy several times a week or more, a proportion that exceeds the average of the overall cohort. Candy consumption has previously been identified as an independent risk factor in our trial [17].

Acevedo-Garcia et al. designed a cross-national framework that integrates social epidemiology into immigrant health research. Factors from the sending country (i.e., the country of origin) may have an impact on immigrant health before and immigration, and continue to do so throughout their life, either individually or in combination with factors from the receiving country (i.e., the country of destination) [34].

Comparisons of the country-of-origin categories in our study yielded large differences in education level, especially in proportions of mothers with an education of ≤ 9 years. Among the non-European country categories, 32%–52% of the mothers had ≤ 9 years of education, while among European country categories, proportions were 8.3%–11%. The education level may be a factor that reflects schooling in the country of origin. In average, 90% of the 25–64-year-olds living in Sweden have reached a level of education equivalent to upper secondary education (9 years) [35]. Education level has previously been identified as an independent risk factor for dental caries, both in our trial and in other studies [17, 36]. The non-European country category also stood out in terms of family income, with nearly 60% placing at the lowest family income level. Candy consumption was common across the entire cohort: only 9.7% of 3-year-olds reported never eating candy, and 62% consumed it about once a week. This pattern may reflect Sweden’s tradition of eating candy primarily on Saturdays. However, considering the WHO guidelines on free sugar intake [37], and the fact that about one third of participants reported daily consumption of sugar-containing beverages, it is likely that many children had exceeded the recommended levels of free sugar of 5%–10% of total daily energy intake.

In Sweden, sales of sugary products remain high [38]. A global report also shows that Western European countries have a higher daily energy intake from sugar-sweetened beverages per person compared to regions such as Sub-Saharan Africa and the Asia-Pacific [39].

Moving from these regions to a country with higher sugar consumption may increase exposure to, and intake, of these products. This suggests that the cohort is generally exposed to a high availability of products with added sugar, which could have contributed to the overall development of dental caries observed in the group.

Risk of confounding factors is always present in observational studies. We included a regression model that addressed dependence and adjusted for potential confounding factors. Besides the ones studied, we found several other factors associated with increased caries development. The floor effect was treated in the statistical model. The classification of languages into countries and regions, as mentioned above, has several risks of bias that would then affect the determination of income level. Some children had several non-Swedish home languages spoken at home; in these children, the classification was based on the language associated with the lower-income category. Further, some languages are spoken in several countries or regions around the world; for example, English is spoken in the United Kingdom, the United States, Australia, and other countries. Similarly, some languages are spoken across neighboring countries or areas, making it difficult to associate the language with a single country. For instance, Arabic is spoken in several countries throughout the Middle East and North Africa. Additionally, some minority languages in Sweden, like Finnish, are also spoken in Finland. Although we had no further information regarding the family’s history, important information related to caries development was found in the Mixed category, as well as in the regional and income classifications. As the exposure was based on language classification, some degree of misclassification may have occurred, which should be considered when interpreting the observed associations.

The study was a secondary analysis of data collected for the randomized SCS trial, and the weaknesses of that trial have already been discussed elsewhere [16]. Any potential bias from the two prevention programs tested [16], should not have affected the present study and, thus, were not considered in our data analysis. Loss to follow-up might have introduced a risk of selection bias. Despite several sources of bias, it seems highly unlikely that they had any significant effect on our findings.

It must also be mentioned that these are results from a socioeconomic disadvantaged area in Stockholm, Sweden. However, such areas are not unique to Sweden, Europe, or the rest of the world. Nevertheless, local conditions may differ, which can influence the representativeness of our findings. In areas with lower caries risk profiles, the same outcome should probably not be expected.

All these findings show the complexity of interacting factors in multicultural low-socioeconomic areas and the many aspects we need to consider when we try, with interventions, to level out inequalities in oral health. As found in the Granlund et al., life course factors may play a role [33]. Participation in preventive measures and access to subsidized dental care, including free dental care for children, may have a positive impact on caries development in the next generation. However, we would like to find a way that shortens this transitional phase, while working for the long-term good.

We can review our structures regarding how to make it easier for newcomers to find their way around the dental care system, and to improve understanding of preventive messages. Such steps, like using interpreters [40], translating oral health education materials into multiple languages [41], and understanding that home visit programs can improve health literacy among those who need an interpreter to fully take part of the information, would be beneficial for reducing the occurrence of caries among young children [42].

Many suggestions for interventions could be made [43], but as the situation is multifaceted, it requires a more tailored approach than is necessary for the general population and may necessitate several approaches. In a qualitative interview study conducted in disadvantaged areas of the Netherlands, parents expressed that the solution to poor oral health was not simply reducing the amount of candy children eat and brushing their teeth; the cause of the oral health issues was more complex. The authors concluded that parental insights are needed to design an effective intervention and that it is important to collaborate with families and prioritize their perspectives and situations [44].

We also need to be aware of the importance of taking local initiatives into consideration and of modifying our approach as new findings come to light. Efforts are needed to ensure the availability of healthy foods in local communities at affordable prices. Lower food expenditure [45] is one of several factors influencing the consumption patterns [46].

Politicians play a key role in up-stream actions. In a global perspective, Swedish dental health is good (3). But this has not always been the case. Early in the 19th century, teachers played an important role in highlighting how poor dental health disrupted the teaching and learning of the students. This led to school dental care being introduced. Child dental care continued to expand, and in 1974, the Dental Care Reform introduced free dental healthcare for children and adolescents [47].

Although recent efforts in Sweden to reduce inequalities in oral health [16, 48, 49] did not yield the expected additional preventive effect on caries development, it remains crucial to continue pursuing these initiatives. This study highlights the connection between immigrant background and the increased risk of developing dental caries, emphasizing the multifaceted issues that may arise with transnational migration. As history clearly demonstrates, the long-term benefits of government support for children’s dental health, including preventive approaches and continuous national monitoring of oral health [50, 51] are beneficial and should be maintained.

As a conclusion, immigrant background was identified as a sociodemographic determinant, with variation in caries outcomes across different groups. Preventive efforts should continue, but these approaches may require further development to better address the specific needs of multicultural and low‑socioeconomic areas. Measures such as increased use of interpreters, multilingual educational materials, and community outreach programs could support families in navigating the dental care system and adopting preventive oral‑health practices.

## Supplementary Information

Supplementary Material 1.

Supplementary Material 2.

## Data Availability

The datasets analyzed in the current study are available from the corresponding author on reasonable request.
