# Supplementary material for: Immigrant background as a determinant of caries development in Swedish children
Source: BMC Oral Health. 2026 Sep 29;26:1994. doi: 10.1186/s12903-026-09919-7 (PMC13663160; doi:10.1186/s12903-026-09919-7)
Supplement: Supplementary file 1 [file 12903_2026_9919_MOESM1_ESM.docx]

**Supplement 1**.

**Table S1.1** The five Region and Income Classification categories used in this study. Income classifications according to the World Bank Country and Lending Groups were used (24).

| High income European country | Middle income European country | High income non-European country | Middle income non-European country | Low income non-European country |
| --- | --- | --- | --- | --- |
| Europe & (Central Asia )& North America | Europe & Central Asia | East Asia & Pacific | East Asia & Pacific | Sub-Sahara Africa |
| EU 15/North America /Nordic region | Albanian-Albania | Cantonese-China | Filipino-Philippines | Amharic-Ethiopia |
| Croatian-Croatia | Bosnian-Bosnia and Herzegovina | Chinese-China | Indonesian-Indonesia | Bantu-Tanzania |
| Danish-Denmark | Bulgarian-Bulgaria | Japanese-Japan | Tagalog- Philippines | Gambian English-Gambian Republic |
| Dutch-Netherlands | Macedonian- Republic of North | Mandarin-China | Thai-Thailand | Kirundi-Burundi |
| English-United Kingdom, other countries* | Macedonia | Uyghur-China | Europe & Central Asia | Lingala-Congo |
| Estonian-Estonia | Montenegrin-Montenegro |  | Armenian-Armenia | Mandinka-Senegal and other countries |
| Finnish-Finland/Sweden (minority language) | Russian-Russia |  | Kurdish-multiple countries | Somali-Somalia |
| French-France | Serbian-Serbia |  | Mongolian-Mongolia | Soninke-Mali and other countries |
| German-Germany | Serbo-Croatian |  | Turkish-Turkey | Swahili-Tanzania |
| Greek-Greece | Ukrainian-Ukraine |  | Turkmen-Turkmenistan | Tigrignia-Eritrea |
| Hungarian-Hungary |  |  | Uzbek-Uzbekistan | Wolof-Mauritania and other countries |
| Icelandic-Iceland |  |  | Vietnamese-Vietnam | Middle East & North Africa |
| Italian-Italy |  |  | Sub-Sahara Africa | Dari-Afghanistan |
| Latvian-Latvia |  |  | Afrikaans-South Africa | Pashto-Afghanistan and other countries |
| Lithuanian-Lithuania |  |  | Akan/Twi -Ghana |  |
| Norwegian-Norway |  |  | Berber-Algeria and other countries |  |
| Polish-Poland |  |  | Fula |  |
| Portuguese-Portugal |  |  | Igbo-Nigeria |  |
| Romani-mutiple countries/Sweden |  |  | Kikuyo |  |
| (minority language) |  |  | Yoruba-Nigeria |  |
| Romanian-Romania |  |  | South Asia |  |
| Spanish-Spain |  |  | Bengali-Bangladesh |  |
| Swedish-Sweden** |  |  | Gujarati-India |  |
| * Norh American countries, |  |  | Hindi-India |  |
| Austraila, etc. (not Europe) |  |  | Nepali-Nepal |  |
| ** outside the high-income |  |  | Punjabi-India |  |
| Euorpean countries in the analysis |  |  | Rohingya-Mayanmar and other countries |  |
|  |  |  | Sinhala-Sri Lanka |  |
|  |  |  | Tamil-Sri Lanka and other countries |  |
|  |  |  | Telugu-India |  |
|  |  |  | Urdu-Paksitan |  |
|  |  |  | Middle East & North Africa |  |
|  |  |  | Arabic-multiple countries |  |
|  |  |  | Aramaic |  |
|  |  |  | Assyrian |  |
|  |  |  | Chaldean |  |
|  |  |  | Persian-Iran |  |
|  |  |  | Suryoyo |  |
|  |  |  | Syriac |  |
