# Supplementary material for: Immigrant background as a determinant of caries development in Swedish children
Source: BMC Oral Health. 2026 Sep 29;26:1994. doi: 10.1186/s12903-026-09919-7 (PMC13663160; doi:10.1186/s12903-026-09919-7)
Supplement: Supplementary file 2 [file 12903_2026_9919_MOESM2_ESM.docx]

**Supplement 2, tables S2.1-S2.8**

**Table S2.1** Results from a mixed-effects zero-inflated negative binomial model with a person-specific random intercept for the longitudinal analysis of the outcome ICDAS II ≥ 1. The exposure variable included three categories. The model was adjusted for candy consumption and sugar-containing beverages. Regression coefficients (β), standard errors (SE), and corresponding p-values are presented.

| **Fixed effects model** |  |  |  |
| --- | --- | --- | --- |
|  | Value | Std. | p-value |
|  |  | Error |  |
| (Intercept) | -5.5920 | 0.1975 | <0.0001 |
| Dummy-No Swedish | 1.8964 | 0.2094 | <0.0001 |
| Dummy-Mixed | 1.8048 | 0.2447 | <0.0001 |
| Time | 0.7728 | 0.0519 | <0.0001 |
| Dummy-Candy consumption level 2 | -0.1064 | 0.0293 | 0.0003 |
| Dummy-Candy consumption level 3 | 0.0775 | 0.0357 | 0.0302 |
| Dummy-Candy consumption level 4 | 0.3646 | 0.0574 | <0.0001 |
| Dummy-Tooth brushing | -0.1537 | 0.0302 | <0.0001 |
| Dummy-Consumption of sugar-containing beverages level 2 | 0.3078 | 0.0312 | <0.0001 |
| Dummy-Consumption of sugar-containing beverages level 3 | 0.2415 | 0.0400 | <0.0001 |
| Dummy-Consumption of sugar-containing beverages level 4 | 0.4782 | 0.0409 | <0.0001 |
| Dummy-Consumption of sugar-containing beverages level 5 | 0.4433 | 0.0431 | <0.0001 |
| Dummy-No Swedish*time | -0.2053 | 0.0526 | 0.0001 |
| Dummy-Mixed*time | -0.3192 | 0.0574 | <0.0001 |
| **Zero-inflation model** |  |  |  |
| (Intercept) | 1.9986 |  |  |
| Dummy-No Swedish | -0.3614 |  |  |
| Dummy-Mixed | -0.1166 |  |  |
| time | -1.1440 |  |  |

**Table S2.2.** Results from a mixed-effects zero-inflated negative binomial model with a person-specific random intercept for the longitudinal analysis of the outcome *ICDAS II ≥ 1*. The exposure variable included three categories. The model was adjusted for maternal education and household income. Regression coefficients with standard errors and corresponding *p*-values are presented.

| **Fixed effects model** |  |  |  |
| --- | --- | --- | --- |
|  | Value | Std.Error | p-value |
| (Intercept) | -4.7382 | 0.2551 | <0.0001 |
| Dummy-No Swedish | 1.7159 | 0.2271 | <0.0001 |
| Dummy-Mixed | 1.6912 | 0.2554 | <0.0001 |
| time | 0.7533 | 0.0525 | <0.0001 |
| Dummy-Mother’s education level 2 | -0.0507 | 0.1549 | 0.7434 |
| Dummy-Mother’s education level 3 | -0.4724 | 0.1650 | 0.0042 |
| Dummy-Family monthly income level 2 | -0.5327 | 0.1408 | 0.0002 |
| Dummy-Family monthly income level 3 | -0.5982 | 0.1669 | 0.0003 |
| Dummy-No Swedish*time | -0.2358 | 0.0539 | <0.0001 |
| Dummy-Mixed*time | -0.3434 | 0.0590 | <0.0001 |
| **Zero-inflation model** |  |  |  |
| (Intercept) | 2.0835 |  |  |
| Dummy-No Swedish | -0.3701 |  |  |
| Dummy-Mixed | -0.0549 |  |  |
| time | -1.2122 |  |  |

**Table S2.3.** Results from a mixed-effects zero-inflated negative binomial model with a person-specific random intercept for the longitudinal analysis of ICDAS II ≥ 1. The exposure variable comprised six categories. Regression coefficients (β), standard errors (SE), and corresponding p-values are presented. Predicted annual means in caries count for the different exposure groups are also shown.

| **Fixed effects model** |  |  |  |
| --- | --- | --- | --- |
|  | Value | Std. | p-value |
|  |  | Error |  |
| (Intercept) | -5.3329 | 0.1896 | <0.0001 |
| Dummy-Low income non-European country | 1.8147 | 0.2571 | <0.0001 |
| Dummy-Middle income non-European country | 1.7920 | 0.2096 | <0.0001 |
| Dummy-High income non-European country | 2.7248 | 0.5841 | <0.0001 |
| Dummy-Middle income European country | 1.7820 | 0.3254 | <0.0001 |
| Dummy-High income European country | 1.7537 | 0.2462 | <0.0001 |
| time | 0.7059 | 0.0493 | <0.0001 |
| Dummy-Low income non-European country*time | -0.1429 | 0.0580 | 0.0138 |
| Dummy-Middle income non-European country*time | -0.1834 | 0.0511 | 0.0003 |
| Dummy-High income non-European country*time | -0.5091 | 0.0781 | <0.0001 |
| Dummy-Middle income European country*time | -0.0462 | 0.0640 | 0.4709 |
| Dummy-High income European country*time | -0.3446 | 0.0574 | <0.0001 |
| **Zero-inflation model** |  |  |  |
| (Intercept) | 2.0222 |  |  |
| Dummy-Low income non-European country | -0.1303 |  |  |
| Dummy-Middle income non-European country | -0.4362 |  |  |
| Dummy-High income non-European country | -1.4260 |  |  |
| Dummy-Middle income European country | -0.0743 |  |  |
| Dummy-High income European country | -0.0279 |  |  |
| time | -1.1700 |  |  |
| **Predicted mean caries count in the different groups** | |  |  |
| hilo | time | predicted |  |
| Dummy-Low income non-European country | 1 | 0.0170 |  |
| Dummy-Low income non-European country | 2 | 0.0558 |  |
| Dummy-Low income non-European country | 3 | 0.1340 |  |
| Dummy-Middle income non-European country | 1 | 0.0194 |  |
| Dummy-Middle income non-European country | 2 | 0.0561 |  |
| Dummy-Middle income non-European country | 3 | 0.1213 |  |
| Dummy-High income European country | 1 | 0.0122 |  |
| Dummy-High income European country | 2 | 0.0336 |  |
| Dummy-High income European country | 3 | 0.0676 |  |
| Dummy-Middle income European country | 1 | 0.0175 |  |
| Swedish | 1 | 0.0029 |  |
| Swedish | 2 | 0.0115 |  |
| Swedish | 3 | 0.0327 |  |
| Dummy-Middle income European country | 2 | 0.0641 |  |
| Dummy-Middle income European country | 3 | 0.1717 |  |
| Dummy-High income non-European country | 1 | 0.0574 |  |
| Dummy-High income non-European country | 2 | 0.0930 |  |

**Table S2.4.** Results from a mixed-effects zero-inflated negative binomial model with a person-specific random intercept for the longitudinal analysis of the outcome ICDAS II ≥ 1. The exposure variable included three categories. The model was adjusted for candy consumption and sugar-containing beverages. Regression coefficients (β), standard errors (SE), and corresponding p-values are presented.

| **Fixed effects model** |  |  |  |
| --- | --- | --- | --- |
|  | Value | Std.Error | p-value |
| (Intercept) | -5.4717 | 0.1914 | <0.0001 |
| Dummy-Low income non-European country | 1.8399 | 0.2562 | <0.0001 |
| Dummy-Middle income non-European country | 1.7236 | 0.2096 | <0.0001 |
| Dummy-High income non-European country | 2.4815 | 0.5805 | <0.0001 |
| Dummy-Middle income European country | 1.4179 | 0.3272 | <0.0001 |
| Dummy-High income European country | 1.6546 | 0.2463 | <0.0001 |
| time | 0.7258 | 0.0496 | <0.0001 |
| Dummy-Candy consumption level 2 | -0.1294 | 0.0291 | <0.0001 |
| Dummy-Candy consumption level 3 | 0.0489 | 0.0357 | 0.1705 |
| Dummy-Candy consumption level 4 | 0.3642 | 0.0574 | <0.0001 |
| Dummy-Tooth brushing | -0.1017 | 0.0303 | 0.0008 |
| Dummy-Consumption of sugar-containing beverages level 2 | 0.3038 | 0.0311 | <0.0001 |
| Dummy-Consumption of sugar-containing beverages level 3 | 0.2403 | 0.0398 | <0.0001 |
| Dummy-Consumption of sugar-containing beverages level 4 | 0.4743 | 0.0408 | <0.0001 |
| Dummy-Consumption of sugar-containing beverages level 5 | 0.4152 | 0.0429 | <0.0001 |
| Dummy-Low income non-European country*time | -0.1403 | 0.0575 | 0.0148 |
| Dummy-Middle income non-European country*time | -0.1683 | 0.0506 | 0.0009 |
| Dummy-High income non-European country*time | -0.4147 | 0.0777 | <0.0001 |
| Dummy-Middle income European country*time | 0.0732 | 0.0653 | 0.2625 |
| Dummy-High income European country*time | -0.3127 | 0.0572 | <0.0001 |
| **Zero-inflation model** |  |  |  |
| (Intercept) | 1.9655 |  |  |
| Dummy-Low income non-European country | -0.1489 |  |  |
| Dummy-Middle income non-European country | -0.4192 |  |  |
| Dummy-High income non-European country | -1.5647 |  |  |
| Dummy-Middle income European country | -0.0952 |  |  |
| Dummy-High income European country | -0.0442 |  |  |
| time | -1.1343 |  |  |

**Table S2.5.** Results from a mixed-effects zero-inflated negative binomial model with a person-specific random intercept for the longitudinal analysis of the outcome *ICDAS II ≥ 1*. The exposure variable included six categories. The model was adjusted for maternal education and household income. Regression coefficients with standard errors and corresponding *p*-values are presented.

| **Fixed effects model** |  |  |  |
| --- | --- | --- | --- |
|  | Value | Std.Error | p-value |
| (Intercept) | -4.6616 | 0.2533 | <0.0001 |
| Dummy-Low income non-European country | 1.5621 | 0.2786 | <0.0001 |
| Dummy-Middle income non-European country | 1.5691 | 0.2275 | <0.0001 |
| Dummy-High income non-European country | 2.6500 | 0.5903 | <0.0001 |
| Dummy-Middle income European country | 1.7619 | 0.3343 | <0.0001 |
| Dummy-High income European country | 1.6353 | 0.2571 | <0.0001 |
| time | 0.7503 | 0.0501 | <0.0001 |
| Dummy-Mother’s education level 2 | -0.0614 | 0.1579 | 0.6976 |
| Dummy-Mother’s education level 3 | -0.4624 | 0.1700 | 0.0066 |
| Dummy-Family monthly income level 2 | -0.5497 | 0.1417 | 0.0001 |
| Dummy-Family monthly income level 3 | -0.6597 | 0.1667 | 0.0001 |
| Dummy-Low income non-European country*time | -0.1985 | 0.0590 | 0.0008 |
| Dummy-Middle income non-European country*time | -0.2255 | 0.0519 | <0.0001 |
| Dummy-High income non-European country*time | -0.5541 | 0.0790 | <0.0001 |
| Dummy-Middle income European country*time | -0.0906 | 0.0649 | 0.1627 |
| Dummy-High income European country*time | -0.3620 | 0.0584 | <0.0001 |
| **Zero-inflation model** |  |  |  |
| (Intercept) | 2.0026 |  |  |
| Dummy-Low income non-European country | -0.1238 |  |  |
| Dummy-Middle income non-European country | -0.4206 |  |  |
| Dummy-High income non-European country | -1.3913 |  |  |
| Dummy-Middle income European country | -0.0224 |  |  |
| Dummy-High income European country | 0.0101 |  |  |
| time | -1.1869 |  |  |

**Seven language categories**

**Table S2.6.** Results from a mixed-effects zero-inflated negative binomial model with a person-specific random intercept for the longitudinal analysis of ICDAS II ≥ 1. The exposure variable comprised six categories. Regression coefficients, standard errors, and corresponding p-values are presented. Predicted annual means in caries count for the different exposure groups are also shown.

| **Fixed effects model** |  |  |  |
| --- | --- | --- | --- |
|  | Value | Std.Error | p-value |
| (Intercept) | -5.4219 | 0.1945 | <0.0001 |
| Dummy-Low income non-European country | 1.7184 | 0.2742 | <0.0001 |
| Dummy-Middle income non-European country | 1.9259 | 0.2179 | <0.0001 |
| Dummy-High income non-European country | 3.0477 | 0.6866 | <0.0001 |
| Dummy-Middle income European country | 1.8994 | 0.3569 | <0.0001 |
| Dummy-High income European country | 2.0003 | 0.2723 | <0.0001 |
| Dummy-Mixed | 1.8004 | 0.2451 | <0.0001 |
| time | 0.7283 | 0.0514 | <0.0001 |
| Dummy-Low income non-European country*time | -0.1368 | 0.0629 | 0.0296 |
| Dummy-Middle income non-European country*time | -0.1824 | 0.0534 | 0.0006 |
| Dummy-High income non-European country*time | -0.5347 | 0.0828 | <0.0001 |
| Dummy-Middle income European country*time | -0.0808 | 0.0689 | 0.2406 |
| Dummy-High income European country*time | -0.3413 | 0.0612 | <0.0001 |
| Dummy-Mixed*time | -0.3117 | 0.0578 | <0.0001 |
| **Zero-inflation model** |  |  |  |
|  |  |  |  |
| (Intercept) | 2.0198 |  |  |
| Dummy-Low income non-European country | -0.1841 |  |  |
| Dummy-Middle income non-European country | -0.5070 |  |  |
| Dummy-High income non-European country | -1.7775 |  |  |
| Dummy-Middle income European country | -0.0403 |  |  |
| Dummy-High income European country | -0.1643 |  |  |
| Dummy-Mixed | -0.0770 |  |  |
| time | -1.1654 |  |  |
| **Predicted mean caries count in the different groups** |  |  |  |
|  | time | predicted |  |
| Dummy-Mixed | 1 | 0.0128 |  |
| Dummy-Mixed | 2 | 0.0367 |  |
| Dummy-Mixed | 3 | 0.0770 |  |
| Dummy-Middle income non-European country | 1 | 0.0217 |  |
| Dummy-Middle income non-European country | 2 | 0.0627 |  |
| Dummy-Middle income non-European country | 3 | 0.1371 |  |
| Dummy-High income European country | 1 | 0.0161 |  |
| Dummy-High income European country | 2 | 0.0437 |  |
| Dummy-High income European country | 3 | 0.0874 |  |
| Dummy-Low income non-European country | 1 | 0.0151 |  |
| Dummy-Middle income European country | 1 | 0.0173 |  |
| Dummy-Low income non-European country | 2 | 0.0500 |  |
| Dummy-Low income non-European country | 3 | 0.1221 |  |
| Swedish | 1 | 0.0027 |  |
| Swedish | 2 | 0.0109 |  |
| Swedish | 3 | 0.0320 |  |
| Dummy-Middle income European country | 2 | 0.0633 |  |
| Dummy-Middle income European country | 3 | 0.1689 |  |
| Dummy-High income non-European country | 1 | 0.0809 |  |
| Dummy-High income non-European country | 2 | 0.1220 |  |
| Dummy-High income non-European country | 3 | 0.1602 |  |

**Table S2.7.** Results from a mixed-effects zero-inflated negative binomial model with a person-specific random intercept for the longitudinal analysis of the outcome *ICDAS II ≥ 1*. The exposure variable included seven categories. The model was adjusted for maternal education and household income. Regression coefficients with standard errors and corresponding *p*-values are presented.

| **Fixed effects model** |  |  |  |
| --- | --- | --- | --- |
|  | Value | Std. | p-value |
|  |  | Error |  |
| (Intercept) | -5.6467 | 0.1975 | <0.0001 |
| Dummy-Low income non-European country | 1.8490 | 0.2737 | <0.0001 |
| Dummy-Middle income non-European country | 1.9345 | 0.2186 | <0.0001 |
| Dummy-High income non-European country | 2.9392 | 0.6824 | <0.0001 |
| Dummy-Middle income European country | 1.6167 | 0.3594 | <0.0001 |
| Dummy-High income European country | 1.9583 | 0.2731 | <0.0001 |
| Dummy-Mixed | 1.8356 | 0.2447 | <0.0001 |
| time | 0.7792 | 0.0519 | <0.0001 |
| Dummy-Candy consumption level 2 | -0.1082 | 0.0296 | 0.0003 |
| Dummy-Candy consumption level 3 | 0.0607 | 0.0361 | 0.0928 |
| Dummy-Candy consumption level 4 | 0.3565 | 0.0575 | <0.0001 |
| Dummy-Tooth brushing | -0.1024 | 0.0306 | 0.0008 |
| Dummy-Consumption of sugar-containing beverages level 2 | 0.3014 | 0.0314 | <0.0001 |
| Dummy-Consumption of sugar-containing beverages level 3 | 0.2375 | 0.0401 | <0.0001 |
| Dummy-Consumption of sugar-containing beverages level 4 | 0.4744 | 0.0410 | <0.0001 |
| Dummy-Consumption of sugar-containing beverages level 5 | 0.4293 | 0.0433 | <0.0001 |
| Dummy-Low income non-European country*time | -0.1739 | 0.0628 | 0.0056 |
| Dummy-Middle income non-European country*time | -0.2025 | 0.0534 | 0.0001 |
| Dummy-High income non-European country*time | -0.4848 | 0.0831 | <0.0001 |
| Dummy-Middle income European country*time | 0.0029 | 0.0707 | 0.9669 |
| Dummy-High income European country*time | -0.3296 | 0.0616 | <0.0001 |
| Dummy-Mixed*time | -0.3284 | 0.0574 | <0.0001 |
| **Zero-inflation model** |  |  |  |
| (Intercept) | 1.9570 |  |  |
| Dummy-Low income non-European country | -0.1860 |  |  |
| Dummy-Middle income non-European country | -0.4833 |  |  |
| Dummy-High income non-European country | -1.8491 |  |  |
| Dummy-Middle income European country | -0.0320 |  |  |
| Dummy-High income European country | -0.1537 |  |  |
| Dummy-Mixed | -0.1056 |  |  |
| time | -1.1284 |  |  |

**Table S2.8.** Results from a mixed-effects zero-inflated negative binomial model with a person-specific random intercept for the longitudinal analysis of the outcome *ICDAS II ≥ 1*. The exposure variable included seven categories. The model was adjusted for maternal education and household income. Regression coefficients with standard errors and corresponding *p*-values are presented.

| **Fixed effects model** |  |  |  |
| --- | --- | --- | --- |
|  | Value | Std.Error | p-value |
| (Intercept) | -4.7652 | 0.2580 | <0.0001 |
| Dummy-Low income non-European country | 1.5209 | 0.2972 | <0.0001 |
| Dummy-Middle income non-European country | 1.7099 | 0.2375 | <0.0001 |
| Dummy-High income non-European country | 2.8772 | 0.6921 | <0.0001 |
| Dummy-Middle income European country | 1.8882 | 0.3661 | <0.0001 |
| Dummy-High income European country | 1.8349 | 0.2848 | <0.0001 |
| Dummy-Mixed | 1.7203 | 0.2553 | <0.0001 |
| time | 0.7674 | 0.0523 | <0.0001 |
| Dummy-Mother’s education level 2 | -0.0651 | 0.1578 | 0.6802 |
| Dummy-Mother’s education level 3 | -0.4875 | 0.1692 | 0.0040 |
| Dummy-Family monthly income level 2 | -0.5359 | 0.1419 | 0.0002 |
| Dummy-Family monthly income level 3 | -0.6003 | 0.1678 | 0.0004 |
| Dummy-Low income non-European country*time | -0.2127 | 0.0639 | 0.0009 |
| Dummy-Middle income non-European country*time | -0.2224 | 0.0543 | <0.0001 |
| Dummy-High income non-European country*time | -0.5745 | 0.0836 | <0.0001 |
| Dummy-Middle income European country*time | -0.1222 | 0.0697 | 0.0797 |
| Dummy-High income European country*time | -0.3411 | 0.0624 | <0.0001 |
| Dummy-Mixed*time | -0.3548 | 0.0588 | <0.0001 |
| **Zero-inflation model** |  |  |  |
| (Intercept) | 2.0302 |  |  |
| Dummy-Low income non-European country | -0.1889 |  |  |
| Dummy-Middle income non-European country | -0.4884 |  |  |
| Dummy-High income non-European country | -1.7522 |  |  |
| Dummy-Middle income European country | 0.0054 |  |  |
| Dummy-High income European country | -0.1281 |  |  |
| Dummy-Mixed | -0.0437 |  |  |
| time | -1.1914 |  |  |
